# Artificial intelligence model for automated surgical instrument detection and counting: an experimental proof-of-concept study

**DOI:** 10.1186/s13037-024-00406-y

**Published:** 2024-07-21

**Authors:** Ekamjit S. Deol, Grant Henning, Spyridon Basourakos, Ranveer M. S. Vasdev, Vidit Sharma, Nicholas L. Kavoussi, R. Jeffrey Karnes, Bradley C. Leibovich, Stephen A. Boorjian, Abhinav Khanna

**Affiliations:** 1 Department of Urology, Mayo Clinic, Rochester, MN USA; 2 Department of Urology, Vanderbilt University Medical Center, Nashville, TN USA

**Keywords:** Retained surgical items, Computer vision, Artificial Intelligence, Surgical tool detection, Surgical safety

## Abstract

**Background:**

Retained surgical items (RSI) are preventable events that pose a significant risk to patient safety. Current strategies for preventing RSIs rely heavily on manual instrument counting methods, which are prone to human error. This study evaluates the feasibility and performance of a deep learning-based computer vision model for automated surgical tool detection and counting.

**Methods:**

A novel dataset of 1,004 images containing 13,213 surgical tools across 11 categories was developed. The dataset was split into training, validation, and test sets at a 60:20:20 ratio. An artificial intelligence (AI) model was trained on the dataset, and the model’s performance was evaluated using standard object detection metrics, including precision and recall. To simulate a real-world surgical setting, model performance was also evaluated in a dynamic surgical video of instruments being moved in real-time.

**Results:**

The model demonstrated high precision (98.5%) and recall (99.9%) in distinguishing surgical tools from the background. It also exhibited excellent performance in differentiating between various surgical tools, with precision ranging from 94.0 to 100% and recall ranging from 97.1 to 100% across 11 tool categories. The model maintained strong performance on a subset of test images containing overlapping tools (precision range: 89.6–100%, and recall range 97.2–98.2%). In a real-time surgical video analysis, the model maintained a correct surgical tool count in all non-transition frames, with a median inference speed of 40.4 frames per second (interquartile range: 4.9).

**Conclusion:**

This study demonstrates that using a deep learning-based computer vision model for automated surgical tool detection and counting is feasible. The model’s high precision and real-time inference capabilities highlight its potential to serve as an AI safeguard to potentially improve patient safety and reduce manual burden on surgical staff. Further validation in clinical settings is warranted.

**Supplementary Information:**

The online version contains supplementary material available at 10.1186/s13037-024-00406-y.

## Background

Retained surgical items (RSIs) are surgical instruments or materials unintentionally left inside a patient’s body after surgery [1]. RSIs are considered “never events,” which are defined as serious, preventable incidents that should ideally never occur in healthcare settings [2]. Despite increased efforts to prevent RSIs, they remain a significant problem, with an estimated incidence of 1 in every 3800 surgeries [3]. The impact of RSIs on patients, healthcare providers, and the healthcare system is substantial, including physical and psychological harm to patients, emotional distress for surgeons, and increased healthcare costs [4]. 

Traditional programs for preventing RSIs center around manual counting of surgical items, commonly conducted by nursing staff [5, 6]. However, such programs often require specialized personnel training, and can increase surgical duration [7]. Furthermore, manual counting is subject to human error due to communication breakdowns, time pressure, competing demands, and environmental distractions [6, 8–10]. Current programs recognize the limitations of individual manual surgical counts and seek to use several layers of security to prevent RSIs [6]. Depending on institutional policies these can include the use of technologies such as radiofrequency tags, [11] barcode labels, [12] and radiographic imaging [13]. 

Recent advancements in the artificial intelligence (AI) field of computer vision, which involves training computers to interpret and understand visual information, have shown promise in helping augment current RSI prevention programs [14]. Prior works using AI techniques have largely focused on computer-aided detection of retained surgical items on medical imaging including the use of AI models to detect retained surgical needles, and other retained radiopaque tag labelled iatrogenic objects on X-ray [15–17]. Outside of AI in medical imaging, there is now an increasing focus on real-time object detection in surgical care. Deep-learning based object detection computer vision algorithms have been applied to a wide range of domains in healthcare, such as the determination of whether individuals are wearing appropriate personal protective equipment [18] and the detection of surgical tools with modest accuracy in a proof-of-concept study [19]. 

Given recent improvements in performance and processing efficiency of computer vision techniques, there may be potential to perform an automated surgical tool count in real-time from live-video. Accordingly, in this study, we sought to evaluate the feasibility and performance of a computer vision model for automated surgical instrument detection and counting. By leveraging state-of-the-art object detection algorithms and training on a novel dataset of surgical instruments, we aim to develop a system that can accurately detect and track surgical tools throughout a procedure, serving as a potential AI safeguard against RSIs.

## Methods

### Study Design and setting

We conducted an experimental proof-of-concept study to evaluate the feasibility and performance of a deep learning-based computer vision model for automated surgical tool detection and counting. The study was performed at the Department of Urology, Mayo Clinic, Rochester, Minnesota, USA, between January 2024 and May 2024.

### Hypothesis

We hypothesized that a deep learning-based computer vision model could accurately detect and classify surgical instruments in real-time from a standard surgical table, potentially serving as an AI safeguard against RSIs.

### Primary and secondary outcomes

The primary outcome was the model’s performance in detecting and classifying surgical tools, as measured by precision, recall, and mean average precision, standard measures to benchmark the performance of computer vision models. The secondary outcome was the model’s inference speed (frames processed per second) when applied to real-time surgical video, to assess its suitability for real-world surgical applications.

### Dataset

Following Mayo Clinic Institutional review board approval, we developed a *de novo* dataset consisting of photos of various combinations of commonly used surgical tools, including scalpels, surgical scissors, forceps, hemostats, needle drivers, surgical retractors, surgical skin markers, beakers, syringes, surgical gauzes, and basins. Each image contained several different types of instruments to simulate a real-world surgical tray setup. Each individual tool appeared in several different photos. Each image contained up to four each of scalpels, needle drivers, handheld retractors, forceps, hemostats, and surgical scissors, up to three each of syringes and basins, and two each of beakers and surgical pens, with a maximum of 34 tools per image. We also included variation in tools within categories to recreate the variability seen in real-world surgical settings. Images were captured from various angles to simulate the different views a computer vision system might encounter during a real-world surgical procedure. These included an overtop view (90 degrees from horizontal), 70 degrees above horizontal from front and side views, and 30 degrees above horizontal from the front of the surgical tables. All images were taken on a blue surgical cloth background, and all objects were of surgical grade to ensure the dataset accurately reflected a true surgical environment.

**Fig. 1:**
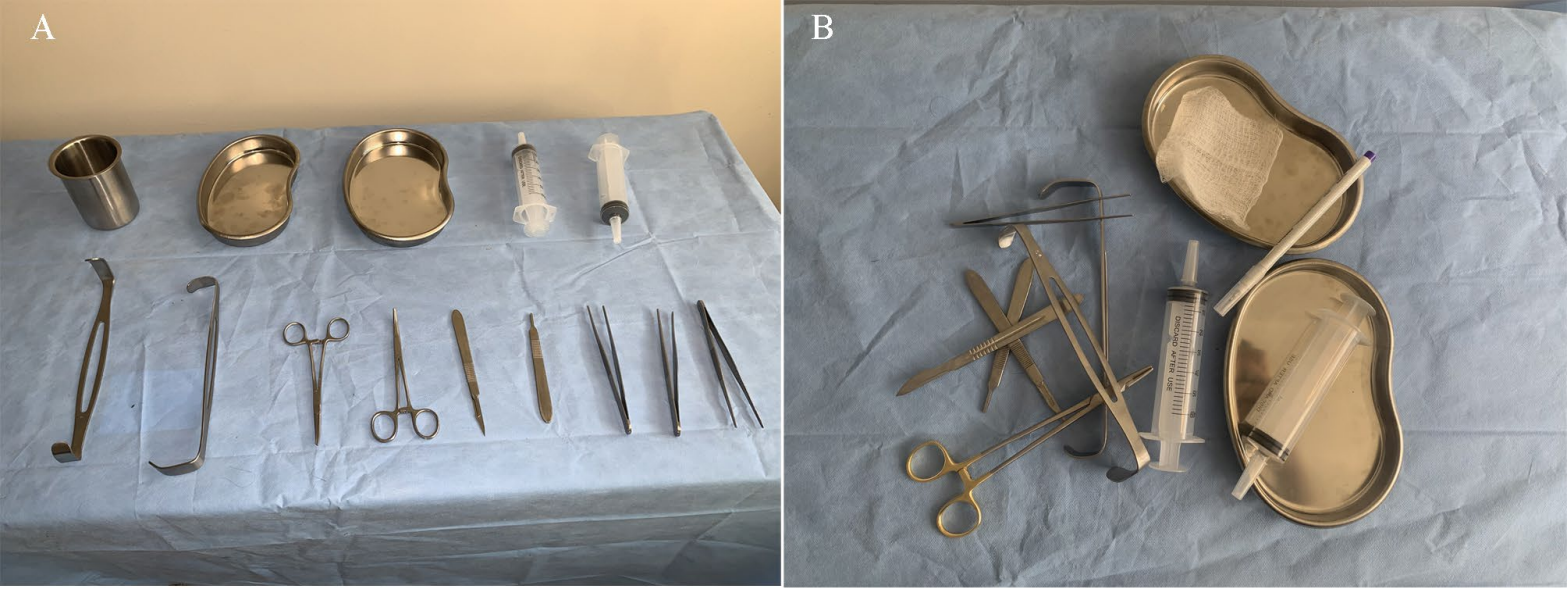
Examples of unlabeled images in surgical tool dataset: **A** – Non-overlapping tools. **B** – Overlapping tools

The full dataset comprised 1004 images, and a total of 13,213 surgical tool instances, including: 1234 scalpel, 814 surgical skin pen, 1304 surgical scissor, 1263 forcep, 2030 hemostat, 1,324 needle driver, 1319 retractor, 676 beaker, 1,187 syringe, 1499 surgical gauze, and 1088 basin instances. In order to evaluate model performance in realistic situations, a subset of 218 images were taken in a cluttered overlapping configuration. Figure 1 highlights examples of non-overlapping and overlapping surgical tool setups. Images were labeled with bounding boxes using the open source Computer Vision Annotation tool [20]. The data was split into training, validation, and test datasets at a 60:20:20 ratio.

Additionally, in order to test the model’s suitability for real-time applications with dynamic instrument exchange, we recorded video footage of various instruments being exchanged in a simulated surgical environment.

### Inclusion and exclusion criteria

Images were included if they contained at least one of the 11 predefined surgical tool categories and were of sufficient quality for annotation. Images were excluded if they were poor quality, did not contain any surgical tools, or contained tools not belonging to the predefined categories.

### Model Architecture and training

For our surgical tool detection model, we utilized the open-source You Only Look Once (YOLO) v9 architecture [21]. YOLOv9 is the latest iteration of the YOLO family of object detection network released in February 2024 [21]. The model employs a Cross Stage Partial Darknet 53 backbone for feature extraction, coupled with a Path Aggregation Network neck for feature aggregation and refinement. The detection head utilizes anchor boxes and a decoupled design to independently handle object scoring, bounding box regression, and class label prediction. We trained the YOLOv9 architecture with 25.5 million parameters on our novel surgical tool dataset. The images were resized to a standard 640 × 640 resolution, and bounding box annotations were normalized to the Common Objects in Context dataset format [22]. Data augmentations such as panning, cropping, brightness adjustment, noise introduction, rotation, horizontal flipping, and cutout were randomly applied to training images to reduce overfitting and improve the model’s robustness. The training was performed on a single NVIDIA V100 graphics processing unit.

### Data analysis

To evaluate the model’s performance, we utilized standard object detection metrics including precision, recall, and mean average precision. In the context of our study, precision indicates the percentage of instances where the model correctly identifies a surgical tool as being present on the table amongst all the surgical tool predictions it makes. Conversely, recall signifies the percentage of surgical tools present in the image that the model successfully identifies as being present on the table. Overall precision and recall were determined for all surgical tools collectively, as well as for each individual surgical tool type. Mean average precision was calculated using a single intersection over union threshold of 0.5 and multiple intersection over union thresholds ranging from 0.50 to 0.95 in intervals of 0.05. To assess the model’s speed and suitability for real-time use during surgery, the model’s frame-by-frame processing time was measured while analyzing a video of surgical instruments being moved in and out of the field of view. This test was used to gauge if the model could keep pace with the dynamic nature of a surgical procedure, correctly identifying tools as they are being used in real-time. All data analysis was conducted in Python using the PyTorch and Ultralytics packages [23]. 

## Results

The overall dataset consisted of 1004 images, of which 603 (7891 tool instances) images were used for model training, 201 (2667 tool instances) for internal validation, and 200 (2655 tool instances) for testing model performance. For detecting the presence or absence of surgical tools in the test dataset the model made 2,693 surgical tool predictions, of which there were 41 instances in which the model falsely identified the background as a surgical tool (false positive). Thus, the overall precision for distinguishing surgical tools from the background was 98.5%. Conversely, the model failed to identify a surgical tool in only three instances, incorrectly labeling a tool as background in all three instances (false negative). This translates to an overall recall (sensitivity) of 99.9%. The model’s mean average precision 50–95 was 88.4%, and mean average precision 50 was 99.4%.

**Fig. 2:**
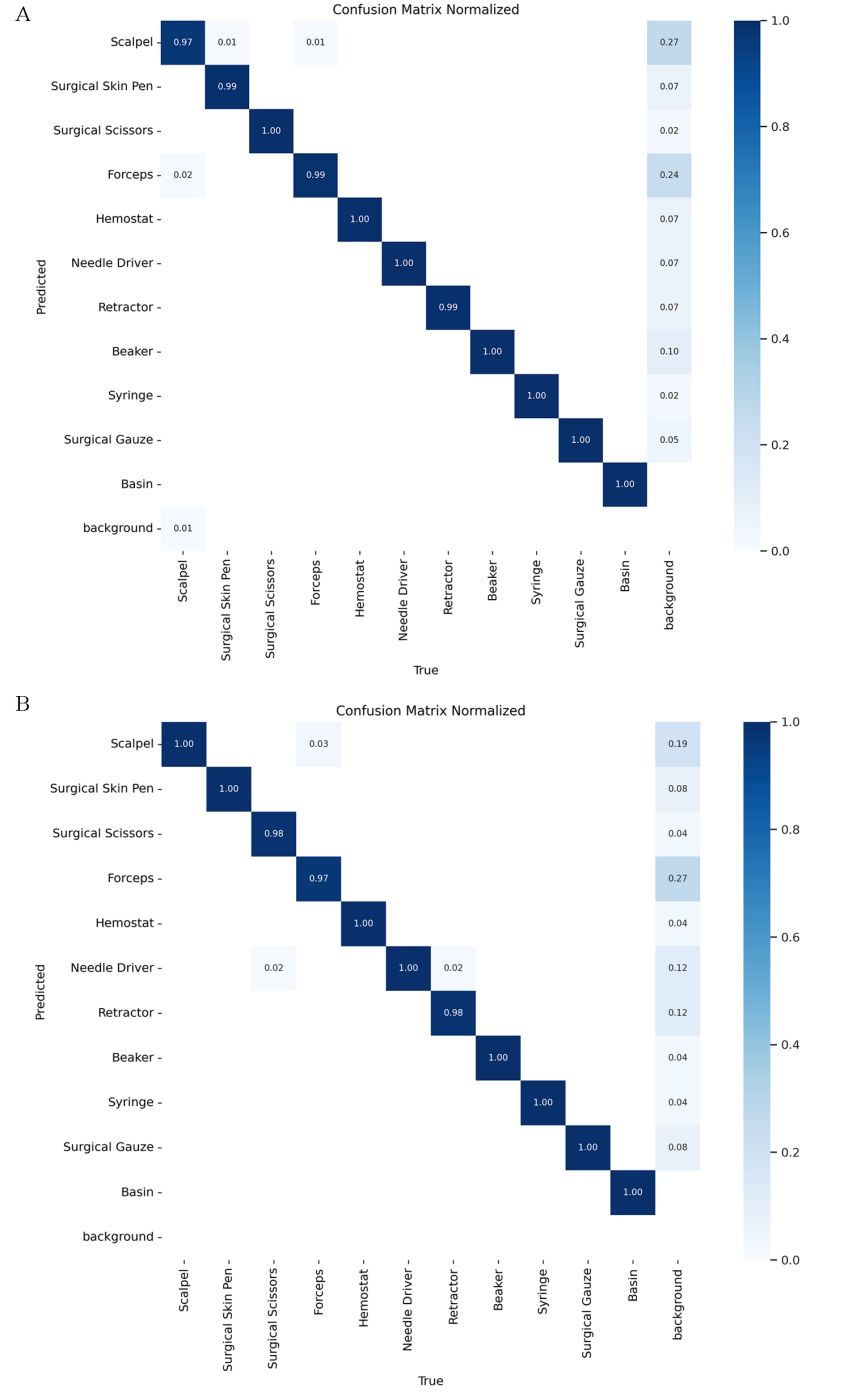
Confusion Matrices showing the model’s performance in classifying surgical instruments. Values represent the proportion of true labels assigned to each predicted class. Correct predictions align along the diagonal, while misclassifications are represented by non-zero values off the diagonal Model performance is shown on **A**. the whole test dataset and **B**. the subset of overlapping surgical tool images only

Model performance was also explored for differentiating between the 11 types of surgical instruments. The basin class exhibited a precision and recall of 100%, indicating that the model perfectly predicted all basin instances without any false positives or false negatives. Syringes also achieved a precision of 99.6% and a recall of 100%, demonstrating nearly perfect performance in identifying all syringe instances. The surgical scissors class attained a precision of 99.2% and a recall of 99.6%. In contrast, the scalpel class had the lowest precision at 94.0% and a recall of 97.1%. The precision and recall values for the remaining instrument classes can be found in Table 1, and a confusion matrix illustrating these results is presented in Fig. 2A.

**Table 1:** Precision and recall values for each of 11 surgical instrument classes

Instrument Class	Precision (%)	Recall (%)
Scalpel	94.02	97.12
Surgical skin pen	98.19	99.39
Surgical scissors	99.24	99.62
Forceps	95.21	98.93
Hemostat	98.60	99.65
Needle driver	97.97	99.66
Retractor	98.88	99.25
Beaker	97.39	100.00
Syringe	99.60	100.00
Surgical gauze	99.26	100.00
Basin	100.00	100.00

**Fig. 3:**
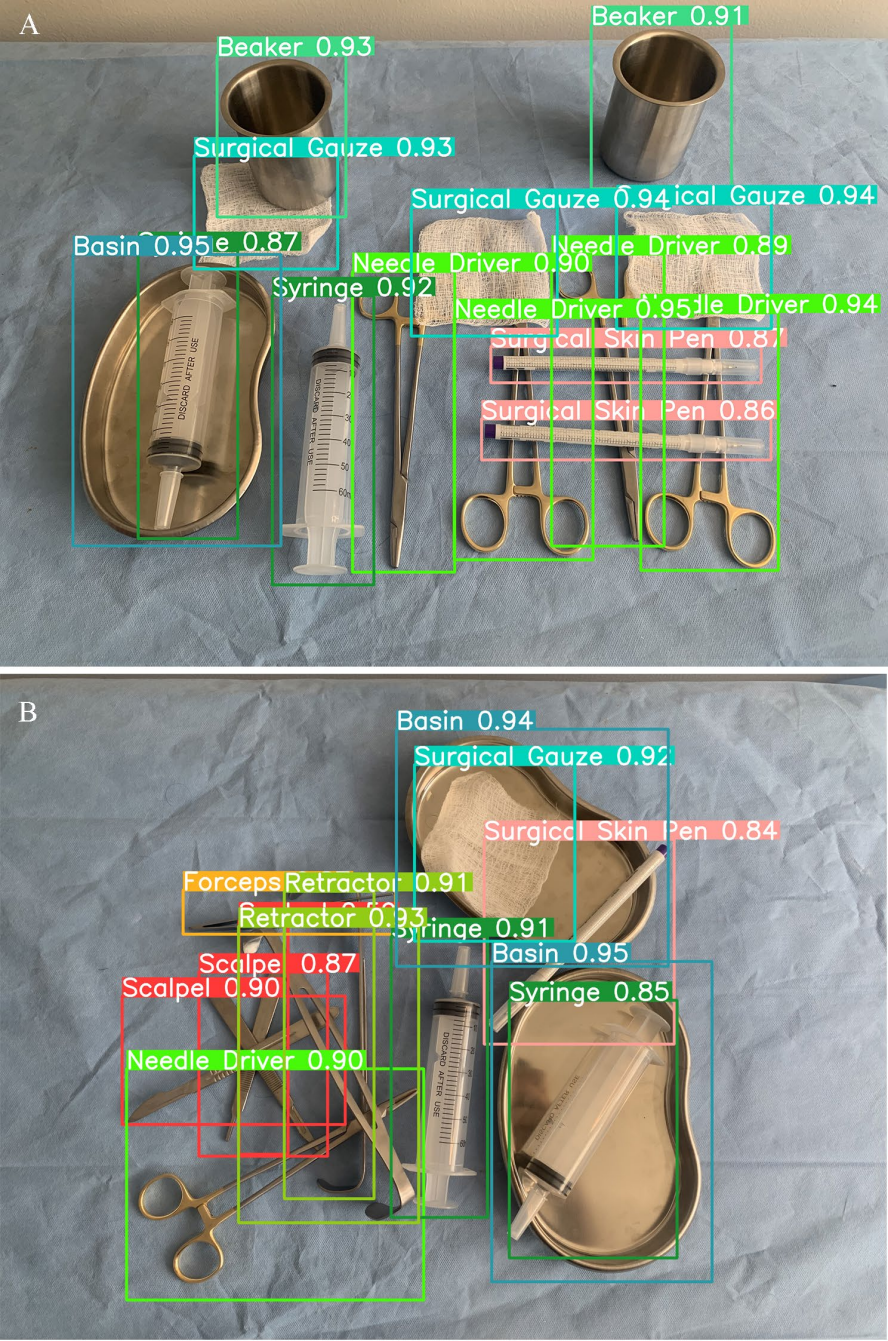
Examples of model generated labels

Similar model performance was observed on the subset of test images containing overlapping tools. For identifying surgical tools from the background, the model achieved a precision of 96.1% and a sensitivity of 100%. Precision remained relatively high when differentiating between surgical tools, ranging from 89.6% for scalpels to 100% for basins. Recall ranged from 97.2% for forceps to 98.2% for retractors. The confusion matrix for the overlapping tool subset is shown in Fig. 2B. Figure 3 shows examples of predictions the model generated.

In a real-time surgical video analysis, the model maintained a correct surgical tool count in all non-transition frames, across tool switches over an hour in a simulated surgery. The model achieved a median inference speed of 24.7 ms (IQR: 3.1 ms) over the 108,128 frames (runtime 1:00:07 h), corresponding to a frame rate of 40.4 FPS (IQR: 4.9) on a single NVIDIA V100. This demonstrates the model’s ability to maintain inference speeds suitable for practical applications in real-world surgery. See Supplementary Table 1.

## Discussion

This study demonstrates the feasibility and effectiveness of employing a deep learning-based computer vision model for the automated detection and enumeration of surgical instruments. The model achieved high precision and recall in distinguishing surgical tools from the background and in differentiating between various surgical instruments, even in challenging scenarios involving overlapping tools. In a real-time surgical video analysis, the model maintained a correct tool count during all non-transition times with an inference speed suitable for real-time use. These results highlight the potential for computer vision models to maintain an automated tool count during surgery which has the potential to reduce errors and thereby help improve surgical safety.

Our study’s high precision and recall in detecting a broad array of surgical tools, even in challenging conditions with overlapping items, address some of the critical gaps in previous research, such as the need for robust detection across a diverse range of surgical objects and the demonstration of an inference speed suitable for practical real-world applications. Lavado et al. previously developed a computer vision model based on YOLOv3 for detecting surgical tools in cluttered trays and performed occlusion reasoning to determine which tool should be removed first following sterilization [24]. Their model was trained on only four different surgical tool classes and performed moderately well (mean average precision at 0.50 of 92.0%). In contrast, our model was trained on 11 different classes and achieved a mean average precision at 0.50 of 99.4%. Also, of note, Lavado et al. photographed surgical tools in a metallic background, whereas our models were trained on tools in a blue surgical cloth background, which is similar to most real-world surgical tray setups [24]. Jiang et al. examined automated surgical tool counting and showed modest model performance (mean average precision 50–95: 88.7%), albeit in a somewhat limited dataset [19]. In contrast, our study builds upon prior literature by using a more diverse dataset with a higher number of tool classes and a greater median number of tools per image, while maintaining similar performance. Further, we are the first to test our model on dynamic video footage in order to demonstrate suitability of our model for real-time inference during live surgery [19]. 

There are notable clinical implications of our findings. In spite of numerous efforts both locally and globally to decrease the incidence of surgical never events, recent data indicates that the frequency of these critical incidents continues to be high [7]. From a systems safety perspective, it is generally believed that most medical errors result from poorly designed systems that allow predictable human mistakes [25]. Accordingly, in an effort to reduce surgical never events, institutions have adopted multi-faceted layered approaches of defense against surgical never events including implementation of standardized safety protocols, [26], regular auditing and monitoring, [27] continuous education and training of surgical staff [7], and a focus on implementational of novel technologies such as radiofrequency tags, [11] radio-labelled tags [12], magnetic surgical instrument location, [28] and a breadth of medical imaging based AI tools [17]. Automated surgical instrument tracking to maintain surgical tool counts throughout the duration of a procedure has the potential to serve as an additional safeguard against RSI. By augmenting or even replacing manual tool counting, an automated AI RSI prevention program could enhance surgical safety while simultaneously allowing surgical personnel to focus on more complex aspects of patient care, thereby improving overall surgical efficiency.

In light of the existing strategies to combat the issue of RSIs, including manual counts and radiofrequency identification systems, our study introduces a computer vision model as an additional layer of verification that has a low threshold to implementation and is highly effective. Analogous to how the integration of additional safeguards such as radiofrequency identification systems can significantly enhance patient safety and reduce RSIs, [29] the incorporation of computer vision models too could further strengthen safety measures against RSIs. Moreover, the ease of integrating computer vision models with current protocols lies in their non-intrusiveness and the relatively low requirement for operational changes. Such computer vision based systems could run in parallel to existing systems, offering real-time feedback without imposing additional tasks on surgical staff, thereby enhancing the overall safety-net against RSIs.

While our study demonstrates the potential of deep learning-based computer vision for automated surgical tool detection and counting, there are several limitations to consider. First, our dataset, although comprehensive and representative of a real surgical environment, may not capture the full range of variability encountered in actual surgical procedures. Factors such as lighting conditions, tool occlusion, and the presence of blood or other bodily fluids could impact model performance in real-world settings. Second, our study focused on a specific set of commonly used surgical tools; the model’s generalizability to less common or specialized instruments remains to be evaluated. Third, while the model achieved strong performance on dynamic video footage, further validation in a clinical setting is necessary to assess its performance under the demands and constraints of actual surgical workflows. Finally, the legal and ethical implications of relying on AI-based systems for patient safety must be carefully considered, including issues of liability, transparency, and the potential for unintended consequences. Despite these limitations, our study provides a strong foundation for future research and development in this area, and the potential benefits of automated RSI prevention systems warrant continued investigation and refinement.

## Conclusion

Our study demonstrates the potential of deep learning and computer vision for automating surgical instrument detection, classification, and occlusion reasoning. The high precision of the model and its occlusion reasoning ability highlight the feasibility of developing comprehensive systems to streamline surgical instrument management and potentially improve patient safety. With further research and validation, such systems could have a significant impact on clinical practice, and may reduce the incidence of RSIs while also reducing the need for surgical personnel to engage in laborious and repetitive manual tasks. To further validate the effectiveness of our algorithm, future research should focus on conducting studies in real-world clinical settings, utilizing larger and more diverse datasets. Finally, the development of standardized publicly available datasets for the development of surgical tool detection algorithms would facilitate more rigorous and comparative studies.

### Electronic supplementary material

Below is the link to the electronic supplementary material.

Supplementary Material 1

## Data Availability

The dataset used and analyzed during the current study is available from the corresponding author (khanna.abhinav@mayo.edu) upon request.
