# Supplementary material for: Artificial intelligence model for automated surgical instrument detection and counting: an experimental proof-of-concept study
Source: Patient Saf Surg. 2024 Jul 21;18:24. doi: 10.1186/s13037-024-00406-y (PMC11265075; doi:10.1186/s13037-024-00406-y)
Supplement: Supplementary file 1 — Supplementary Material 1 [file 13037_2024_406_MOESM1_ESM.docx]

**Supplementary Table 1**: First 10 transitions of simulated surgical video. Full hour long video has tool transitions every ~1-2 minutes:

| Non-transition Frames | Objects Identified During Non-Transition Frames | Ground Truth Objects Present on Frame | Objects Removed or Added during Transition |
| --- | --- | --- | --- |
| 1 to 2186 | 1 Basin, 1 Beaker, 1 Syringe, 1 Hemostat, 1 Retractor, 1 Needle Driver, 1 Surgical Scissors, 1 Forceps, 1 Surgical Skin Pen, 1 Scalpel | 1 Basin, 1 Beaker, 1 Syringe, 1 Hemostat, 1 Retractor, 1 Needle Driver, 1 Surgical Scissors, 1 Forceps, 1 Surgical Skin Pen, 1 Scalpel |  |
| 2224 to 3676 | 1 Basin, 1 Hemostat, 1 Syringe, 1 Beaker, 1 Retractor, 1 Surgical Scissors, 1 Forceps, 1 Surgical Skin Pen, 1 Scalpel | 1 Basin, 1 Hemostat, 1 Syringe, 1 Beaker, 1 Retractor, 1 Surgical Scissors, 1 Forceps, 1 Surgical Skin Pen, 1 Scalpel | Removed Needle Driver |
| 3697 to 6911 | 1 Beaker, 1 Hemostat, 1 Syringe, 1 Retractor, 1 Surgical Scissors, 1 Forceps, 1 Surgical Skin Pen, 1 Scalpel | 1 Beaker, 1 Hemostat, 1 Syringe, 1 Retractor, 1 Surgical Scissors, 1 Forceps, 1 Surgical Skin Pen, 1 Scalpel | Removed basin |
| 6930 to 8741 | 1 Hemostat, 1 Surgical Scissors, 1 Retractor, 1 Beaker, 1 Forceps, 1 Surgical Skin Pen, 1 Scalpel | 1 Hemostat, 1 Surgical Scissors, 1 Retractor, 1 Beaker, 1 Forceps, 1 Surgical Skin Pen, 1 Scalpel | Removed Syringe |
| 8794 to 10828 | 1 Surgical Scissors, 1 Hemostat, 1 Beaker, 1 Forceps, 1 Surgical Skin Pen, 1 Scalpel | 1 Surgical Scissors, 1 Hemostat, 1 Beaker, 1 Forceps, 1 Surgical Skin Pen, 1 Scalpel | Removed retractor |
| 10859 to 12616 | 1 Hemostat, 1 Surgical Scissors, 1 Beaker, 1 Forceps, 1 Scalpel | 1 Hemostat, 1 Surgical Scissors, 1 Beaker, 1 Forceps, 1 Scalpel | Removed skin pen |
| 12667 to 14507 | 1 Surgical Scissors, 1 Beaker, 1 Forceps, 1 Scalpel | 1 Surgical Scissors, 1 Beaker, 1 Forceps, 1 Scalpel | Removed hemostat |
| 14562 to 16415 | 1 Surgical Scissors, 1 Beaker, 1 Forceps | 1 Surgical Scissors, 1 Beaker, 1 Forceps | Removed Scalpel |
| 16448 to 19796 | 1 Surgical Scissors, 1 Beaker | 1 Surgical Scissors, 1 Beaker | Removed Forceps |
| 19808 to 21678 | 1 Surgical Scissors | 1 Surgical Scissors | Removed Beaker |
